# Integrated approaches to miRNAs target definition: time-series analysis in an osteosarcoma differentiative model

**DOI:** 10.1186/s12920-015-0106-0

**Published:** 2015-06-30

**Authors:** A. Grilli, M. Sciandra, M. Terracciano, P. Picci, K. Scotlandi

**Affiliations:** Laboratory of Experimental Oncology, CRS Development of Biomolecular Therapies, Rizzoli Orthopedic Institute, Via di Barbiano 1/10, 40136 Bologna, Italy; PROMETEO, STB, RIT Department, Rizzoli Orthopedic Institute, Bologna, Italy

**Keywords:** microRNA, microRNA target, Array integrations, Osteosarcoma, miR-34a, TGFbeta signaling

## Abstract

**Background:**

microRNAs (miRs) are small non-coding RNAs involved in the fine regulation of several cellular processes by inhibiting their target genes at post-transcriptional level. Osteosarcoma (OS) is a tumor thought to be related to a molecular blockade of the normal process of osteoblast differentiation. The current paper explores temporal transcriptional modifications comparing an osteosarcoma cell line, Saos-2, and clones stably transfected with CD99, a molecule which was found to drive OS cells to terminally differentiate.

**Methods:**

Parental cell line and CD99 transfectants were cultured up to 14 days in differentiating medium. In this setting, OS cells were profiled by gene and miRNA expression arrays. Integration of gene and miRNA profiling was performed by both sequence complementarity and expression correlation. Further enrichment and network analyses were carried out to focus on the modulated pathways and on the interactions between transcriptome and miRNome. To track the temporal transcriptional modification, a PCA analysis with differentiated human MSC was performed.

**Results:**

We identified a strong (about 80 %) gene down-modulation where reversion towards the osteoblast-like phenotype matches significant enrichment in TGFbeta signaling players like AKT1 and SMADs. In parallel, we observed the modulation of several cancer-related microRNAs like miR-34a, miR-26b or miR-378. To decipher their impact on the modified transcriptional program in CD99 cells, we correlated gene and microRNA time-series data miR-34a, in particular, was found to regulate a distinct subnetwork of genes with respect to the rest of the other differentially expressed miRs and it appeared to be the main mediator of several TGFbeta signaling genes at initial and middle phases of differentiation. Integration studies further highlighted the involvement of TGFbeta pathway in the differentiation of OS cells towards osteoblasts and its regulation by microRNAs.

**Conclusions:**

These data underline that the expression of miR-34a and down-modulation of TGFbeta signaling emerge as pivotal events to drive CD99-mediated reversal of malignancy and activation of differentiation in OS cells. Our results describe crucial and specific interacting actors providing and supporting their relevance as potential targets for therapeutic differentiative strategies.

**Electronic supplementary material:**

The online version of this article (doi:10.1186/s12920-015-0106-0) contains supplementary material, which is available to authorized users.

## Background

MicroRNAs (miRNAs) are small non-coding RNAs that act as gene regulators at post-transcriptional level. At present, it is largely established that they have a central role in both physiological and pathological conditions. In particular, miRNAs have a central role in cancer as key regulators of a multitude of processes [1], like cell differentiation [2], cell proliferation, and apoptosis [3]. Their mechanism of action is exerted through the binding of their 6–7 nt seed sequence to 3’UTR of target mRNAs which thus lead to degradation or transcriptional repression, depending on partial or perfect sequence match. Currently, more than 2.5 thousand mature miRNAs have been discovered in humans (miRbase v.20); if we consider the small matching miRNA-target sequence, the entire transcriptome could be a putative target of miRNAs. Several predicting target algorithms, like TargetScan, miRanda, PicTar and Diana MicroT [4–7] are now a routine start point to target definition. However, determination of miRNA-mRNA interactions still remain a difficult task, as these algorithms introduce a very high number of false positives [8]. Furthermore, microRNAs have also been described as positive regulators at transcriptional level of mRNA expression [9–11], with a strict dependency on the cellular context [12]. New methods together with new computational approaches are continuously being developed. Among high-throughput methods, a strategy focused on expression correlation between genes and miRNAs microarrays has been defined [13, 14], based on the evidence that degradation of a mRNA target is preferred to inhibition of its translation [15, 16]. In specific contexts like time-series experiments, integration of miRNA-mRNA expression may add valuable information on dynamic changes in gene regulation with respect to data focused on a single time point. Analysis of differentiative processes by integration of gene and miRNA time-series data may thus result particularly helpful in identifying the set of regulatory interactions at different time-points, and assignment of different microRNAs to specific differentiative phases or processes.

Sarcomas are rare tumors caused by disruptions of mesenchymal cell differentiation [17–19]. In particular, osteosarcoma (OS) is a bone cancer caused by multiple and complex genetic alterations that ultimately result in a blockage of osteoblast differentiation. Although several pathways and genes related to development, such as Wnt signaling [20, 21], TGFbeta signaling [22], Notch signaling [23], Hedgehog signaling [24], have been found to be frequently dysregulated in this sarcoma, a more comprehensive view of the processes that are aberrantly modified during OS differentiation is still missing. Highly regulated expression of genes accomplishes the process of normal osteoblastogenesis during differentiation and development [25]. Considering that OS cells appear to be somehow ‘frozen’ in a state of incomplete osteogenic differentiation [26–28], a better insight into specific gene regulation during OS differentiation may help remove this block and may have therapeutic value. In this paper we investigated integration of time-series miRNome and transcriptome to provide a better comprehension of the potential role that miRNAs may have in reprogramming genome activity coupled with OS differentiation. Recently we found that re-expression of CD99, a cell surface molecule present in osteoblasts but generally lost in OS [29], can inhibit malignancy [29, 30] and reactivate terminal differentiation [28] of OS cells. We took advantage of this experimental model and compared miRNAs-mRNAs interactions of the parental Saos-2 OS cells and osteoblast differentiation-prone Sa/CD99 cell variants at basal and differentiating conditions. Multiple bioinformatics approaches were used: integration of target prediction and expression correlation methods identified modulated genes and pathways that are directly or indirectly under control of miRNAs reprogramming; network visualization clarified differential processes where modulated miRNAs act at each time point; PCA analysis described temporal transcriptional reversion of OS cells towards the osteoblastic phenotype.

## Results

**Fig. 1:**
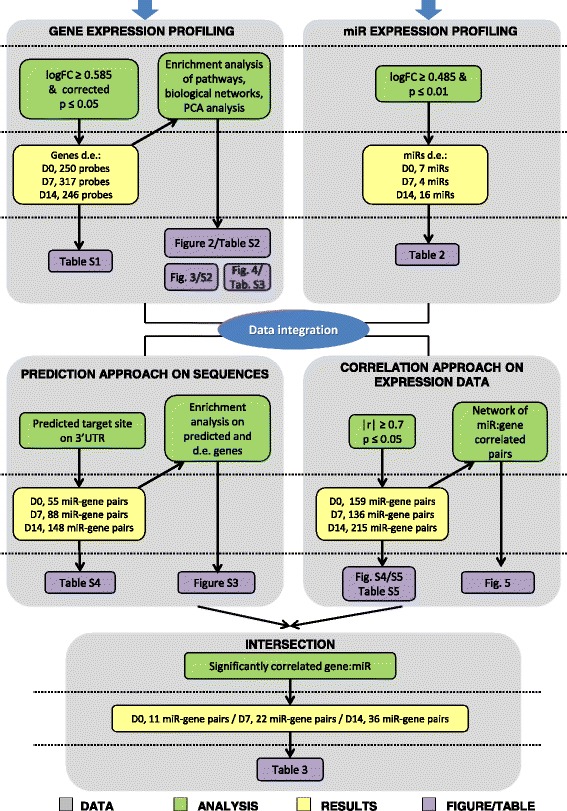
Flowchart of bioinformatic analyses. Gene and miRNA profiling of differentially expressed genes/miRs were initially integrated by correlation score of expression data or by prediction target databases. The two approaches were subsequently integrated to identify most significant miRNAs targets (d.e. = differentially expressed; FC = fold change of absolute values; p = *p*-value; r = Pearson’s product moment correlation coefficient; D0, D7, D14 = respectively day 0, day 7 and day 14)

By Affymetrix GeneChip and miRNA Agilent arrays we compared gene and miRNA expression profiles of CD99-transfected clones *versus* the respective parental cell line; four samples at each time point (two samples for Sa/CD99 and two for Saos-2) were profiled at basal conditions and after 7 and 14 days in osteoblast differentiation medium. A flowchart of the analyses is shown in Fig. 1 and matching numbers of each phase are resumed in Table 1.

**Table 1:** Numbers of bioinformatics analyses for each step are resumed

**Genes differentially expressed**				
	Day 0	Day 7	Day 14	Total
Probes	250	317	246	536
Probes up-regulated (%)	19 (7.6)	52 (16.4)	29 (11.8)	79 (20.1)
Probes down-regulated (%)	231 (92.4)	265 (83.6)	217 (88.2)	315 (79.9)
Annotated genes	159	215	184	350
Genes up-regulated (%)	17 (10.7)	46 (21.4)	24 (13.0)	67 (19.1)
Genes down-regulated (%)	142 (89.3)	169 (78.6)	160 (87.0)	267 (80.9)
**miRs differentially expressed**				
	Day 0	Day 7	Day 14	Total
miRs modulated	7	4	16	22
miRs up-regulated (%)	6 (85.6)	1 (25)	14 (87.5)	16 + 3^a^
miRs down-regulated (%)	1 (14.3)	3 (75)	2 (12.5)	3 + 3^a^
**Correlation and prediction methods**				
	Day 0	Day 7	Day 14	Total
Total correlations	1750	1268	3936	6954
Significant correlations (%)	159 (9.1)	136 (10.7)	215 (5.5)	510 (7.3)
Negative correlations (%)	119 (74.8)	106 (77.9)	70 (32.6)	295 (57.8)
Positive correlations (%)	40 (25.2)	30 (22.1)	145 (67.4)	215 (42.2)
miRs differentially expressed with predicted targets in at least 1 database	7	4	12	18
Total predictions at probe level	96	181	461	738
Total predictions at gene level	55	88	148	216
**Merge of correlation and prediction methods**				
	Day 0	Day 7	Day 14	Total
miRs	2	2	6	9
Genes	11	22	32	50
Significant couples miR-gene	11	22	36	61

^a^3 miRs change their modulation across time points

### CD99 over-expression fosters a massive down-regulation in gene expression of osteosarcoma cells and modulates specific pathways

**Fig. 2:**
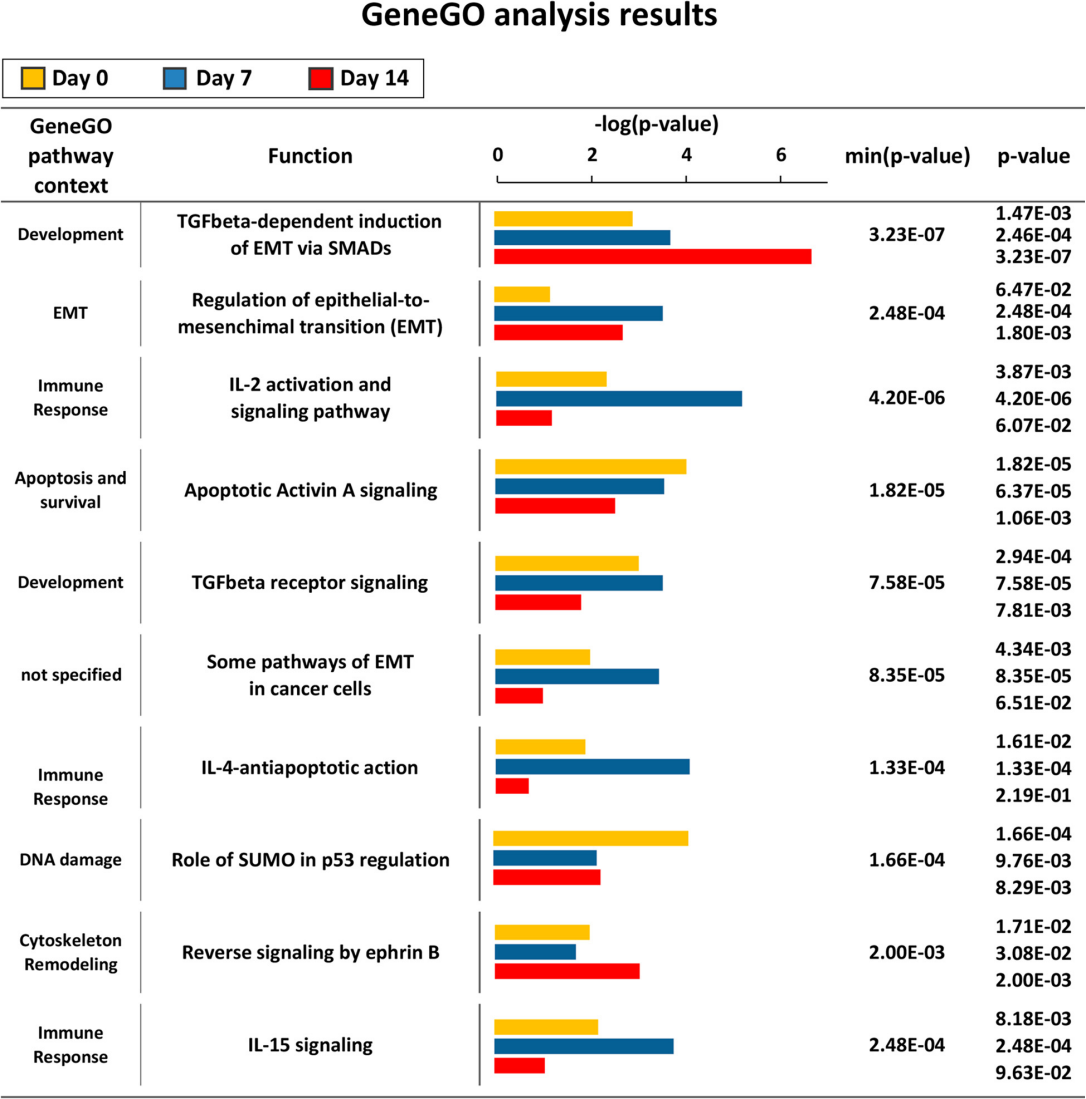
Enrichment analysis of biologically modulated pathways. Analysis first shows a significant enrichment in TGFbeta signaling modulated by SMAD proteins: as the majority of genes are down-regulated, we have inhibition of this pathway in Saos clones, with an effect that increases over time. Other important and significantly modulated are the EMT and Apoptotic pathways. (min (*p*-value) = most significant *p*-value among the 3 time points after multiple correction test)

**Fig. 3:**
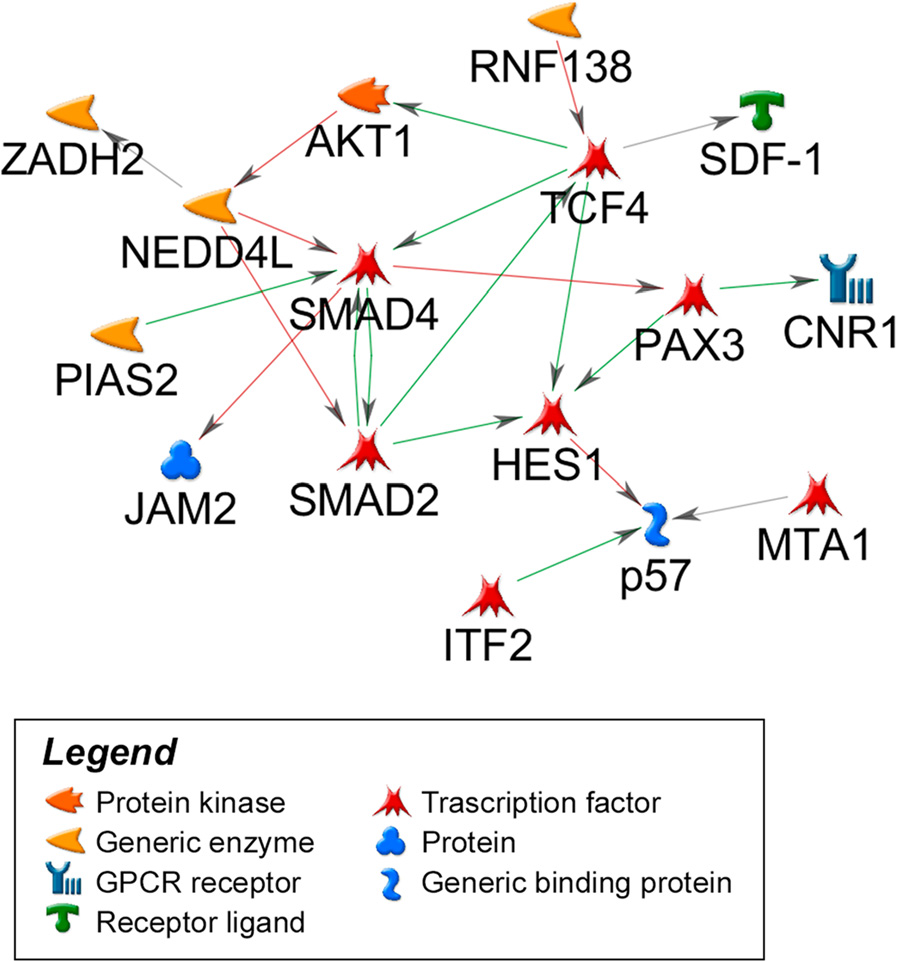
Network analysis of differentially expressed genes at all time points. Among the 64 genes significantly modulated at both basal and differentiative conditions, 16 have direct biological interaction according to literature (positive or activation, in green, negative or inhibition, in red, or unspecified effects, in gray) and participate in the network. All drawn genes are down-regulated

Gene expression analysis identified 250 to 317 probes differentially expressed, depending on the time point (Additional file 1: Table S1). Differentially expressed genes are pointed out in a volcano plot in Additional file 2: Figure S1a. Interestingly, we observed a marked and time-constant down-regulation in gene expression when CD99 is re-expressed and cells are prone to terminal differentiation. From 78 % to 89 % of genes resulted under expressed (|logFC| ≥ 0.585 and *p*-value ≤ 0.05) with respect to the more aggressive and less differentiated parental cell line. Enrichment analysis showed a significant modulation of 32 pathways at day 0, 54 at day 7, and 17 at day 14 (Additional file 3: Table S2 for the 30 most significant pathways). Among the most significant modulated pathways we observed: “*TGF-beta dependent induction of EMT via SMADs*” (*p*-value = 3.78 E-08) and “*Regulation of epithelial to mesenchymal transition*” (*p*-value 2.78 E-06) (Fig. 2). *TGF-beta dependent induction of EMT via SMADs* pathway increased its significance during differentiation, with, over time, an increase of down-regulated genes (i.e., occludin, fibronectin). Genes involved in the apoptotic mechanism were constantly enriched but with wider involvement at initial phases (days 0 and 7), while enrichment in genes regulating the cell cycle (“*regulation of G1/S transition” p*-value = 1.8 E-08) was found only at day 0, in keeping with the functional role of these pathways in the reversion of malignancy and induction of differentiation. Considering single genes, a total of 64 genes were constantly modulated across all differentiative process and all but two constantly down-modulated. These genes were involved in several cellular processes like protection from apoptosis or survival (AKT1, TOX3, SMAD2, BAG5), chemoresistance, (MGST1*)*, Notch pathway (HEY2, and HES1), histone modifications (SNCA) and transcriptional regulation (SMAD4, PAX3). To define network hubs of CD99-mediated differentiation on the 64 genes constantly modulated, a network analysis by GeneGO was performed: 16 genes out of 64 were found to have a direct modulation, with a changeless cascade signaling involving SMAD2 and SMAD4 genes with AKT1 (Fig. 3). Interestingly, there is a variable sequence of gene patterns that interacts with this core network (Additional file 4: Figure S2): (i) at day 0, THEM4, PPP2R5C and C13orf15 (alias RGC32) (ii) at day 7, GAB2, BCL2, GRB10, JAK1 (iii) at day 14, MAPKAPK2, LYN ICAM1, LIF, FN1, HMGA2, VDR, ID1.

**Fig. 4:**
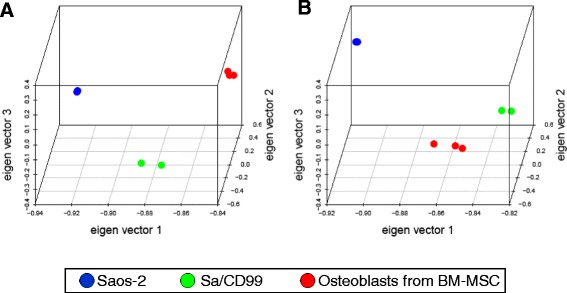
Meta-analysis with human osteoblast cells. Meta-analysis including expression profile of Saos-2, Sa/CD99 cells and human osteoblasts differentiated from bone marrow hMSCs (BM-MSCs) at day 0 (**a**) and day 14 (**b**). Transfected Sa/CD99 cells progressively shift from an OS profile (day 0) towards a less aggressive and more osteoblast-like phenotype (day 14)

PCA meta-analysis with human mesenchymal stem cells (hMSCs) expression data was performed to see CD99 impact on the program of transcriptional differentiation. Due to array platform type and comparable experimental conditions limitations, we only recovered a single dataset of 3 human osteoblast cell lines [31]; these cells were derived from bone marrow hMSCs obtained from iliac crest and directed to osteoblast differentiation [32]. Expression profile of genes differentially expressed at basal (day 0, Fig. 4a and Additional file 5: Table S3) or in differentiative conditions (day 14, Fig. 4b) shows that OS cells prone to have a terminally differentiated phenotype (Sa/CD99 cells) tend to converge to osteoblasts cells. This confirms that our model may reflect a physiological process, thus providing valuable information on reversion toward an osteoblast-like phenotype of OS transfected cells.

### miRNAs modulation during differentiation emerges as a specific mechanism to define changes in gene profiling

Considering that widespread gene down-modulation was observed at all time points in consequence of the re-expression of CD99, we evaluated involvement of miRNAs as a possible epigenetic mechanism of gene regulation. We thus analyzed miRNAs profile in transfectants versus parental cells and identified 7, 4, and 16 miRs with a significant differential expression at days 0, 7, and 14 respectively (Table 2 for miRNAs modulation and Additional file 2: Figure S1b for corresponding volcano plot). At days 0 and 14, the majority of miRs showed an up-regulation (respectively 6/7 and 14/16), in line with the hypothesis that gene expression down-regulation could at least partially be controlled by miRNAs. On the contrary, miR up-regulation is not evident at day 7, maybe because of the lower number of significant miRs. In basal conditions (day 0), up-regulated miRNAs were: miR-1225-3p, miR-1305, miR-1238, miR-425, miR-191* and miR-34a, while miR-378 was the only down-regulated miR. This profile is therefore associated with a less aggressive phenotype more prone to differentiation. Among these miRs only miR-1305 was maintained significantly modulated over osteoblast-like differentiation. Increased expression of miR-34a was significantly up-regulated at days 0 and 7, in keeping with the oncosuppressive role of this miR that negatively regulates cell proliferation while increasing apoptosis [33]. When Sa/CD99 cells are terminally differentiated (day 14) [28], we observed a modulation of a different set of miRs: miR-342-3p was the most down-regulated, while miR-139-3p, miR-1288 and miR-1914* were the most up-regulated.

**Table 2:** Significantly modulated miRNAs in Sa/CD99 vs Saos-2 cells. For each day, log fold change and significance are shown

microRNA	Genomic position	Log fold change			Significance		
		Day 0	Day 7	Day 14	Day 0	Day 7	Day 14
hsa-miR-378	Ch5q32	−0.938	−0.323	0.109	0.00015	*ns*	*ns*
hsa-miR-34a	Ch1p36	0.777	0.781	0.327	0.00177	0.00171	ns
hsa-miR-1225-3p	Ch16p13	1.206	0.142	0.050	0.00193	*ns*	*ns*
hsa-miR-1305	Ch4q34	0.757	−0.965	1.750	0.00466	0.00108	0.00001
hsa-miR-1238	Ch19p13	0.969	0.627	−0.302	0.00593	*ns*	*ns*
hsa-miR-425*	Ch3p21	0.910	0.537	−0.199	000841	*ns*	*ns*
hsa-miR-191*	Ch3p21	1.027	0.553	−0.216	0.00859	*ns*	*ns*
hsa-miR-892b	ChXq27	0.176	−1.047	0.999	*ns*	0.00006	0.00008
hsa-miR-139-3p	Ch11q13	0.024	−1.562	1.013	*ns*	0.00017	0.00289
hsa-miR-500	ChXp11	0.008	−0.249	0.818	*ns*	*ns*	0.00066
hsa-miR-760	Ch1p22	−0.058	−0.251	0.737	*ns*	*ns*	0.00199
hsa-miR-1299	Ch9q21	−0.040	−0.260	0.778	*ns*	*ns*	0.00234
hsa-miR-342-3p	Ch14q32	−0.834	−0.808	−1.018	*ns*	*ns*	0.00428
hsa-miR-1181	Ch19p13	0.045	0.385	0.672	*ns*	*ns*	0.00473
hsa-miR-516a-5p	Ch19q13	−0.001	−0.049	0.512	*ns*	*ns*	0.00529
hsa-miR-1288	Ch17p11	0.575	−0.866	1.269	*ns*	*ns*	0.00547
hsa-miR-150*	Ch19q13	0.177	0.113	0.784	*ns*	*ns*	0.00663
hsa-miR-1914*	Ch20q13	0.276	−0.230	1.040	*ns*	*ns*	0.00669
hsa-miR-26b	Ch2q35	−0.433	−0.443	−0.687	*ns*	*ns*	0.00809
hsa-miR-520e	Ch19q13	0.036	0.231	0.610	*ns*	*ns*	0.00809
hsa-miR-202	Ch10q26	0.007	−0.255	0.607	*ns*	*ns*	0.00882
hsa-miR-1469	Ch15q26	−0.032	−0.146	0.579	*ns*	*ns*	0.00892

*ns* = not significant

### Dynamic interactions between mRNA and miRNA profiles defines miR-34a as a leading player of TGFbeta signaling in CD99 cells

As suggested in recent papers, different high-throughput methods may reduce the number of false positives in miRNA target definition [34]. Thus, we first integrated expression data of genes and microRNAs expression by Pearson’s correlation, then we defined putative target regions in the 3’ UTR of target genes by miRNA-mRNA sequence comparison. For expression correlations, both negative and positive *r* scores were considered. We finally integrated expression correlations and miRNAs target information approaches to identify the most interesting targets of differentially expressed miRs. We only used genes and miRNAs significantly modulated by CD99 transfection instead of calculating correlations or predictions for all or for moderately modulated probe:miRNA couples in arrays [35], since we considered these genes and miRNAs as the most informative for the differentiative program.

Previous expression analysis defined a total of 536 probes and 22 miRs differentially modulated in at least one out of three time points. To determine the putative target genes for each microRNA, we merged the predictions from 4 different prediction target algorithms: Diana MicroT, TargetScan, PicTar and miRanda. We obtained 96, 181, and 461 predicted targets at the 3 time points (Additional file 6: Table S4). There is at least one putative target for each microRNA at days 0 and 7, instead only 12 out of 16 miRs have predicted targets at day 14, because 4 miRs (miR-1181, miR-1288, miR-1914, miR-1469) are missing in the prediction target databases. The analysis showed that mir-34a has the highest number of predicted targets, respectively 44 and 73 genes at days 0 and 7. As expected, several genes are predicted targets of multiple microRNAs. A pathway enrichment analysis by GeneGO on predicted targets was performed to identify the putatively regulated biological pathways. We confirmed in this way the significant enrichment of genes related to TGF-Beta signaling pathway (Additional file 7: Figure S3). Several microRNAs seem to regulate multiple genes of the TGFbeta pathway, like miR-760: SMAD4/SMURF1, miR-378: SMAD2/SMAD4/SOS1, miR-26b: SMAD2/4, miR-520e: AKT1/SMAD4. miR-34a in particular shows a target site on the majority of these (SMAD2, SMAD4, AKT1, SMURF1).

**Fig. 5:**
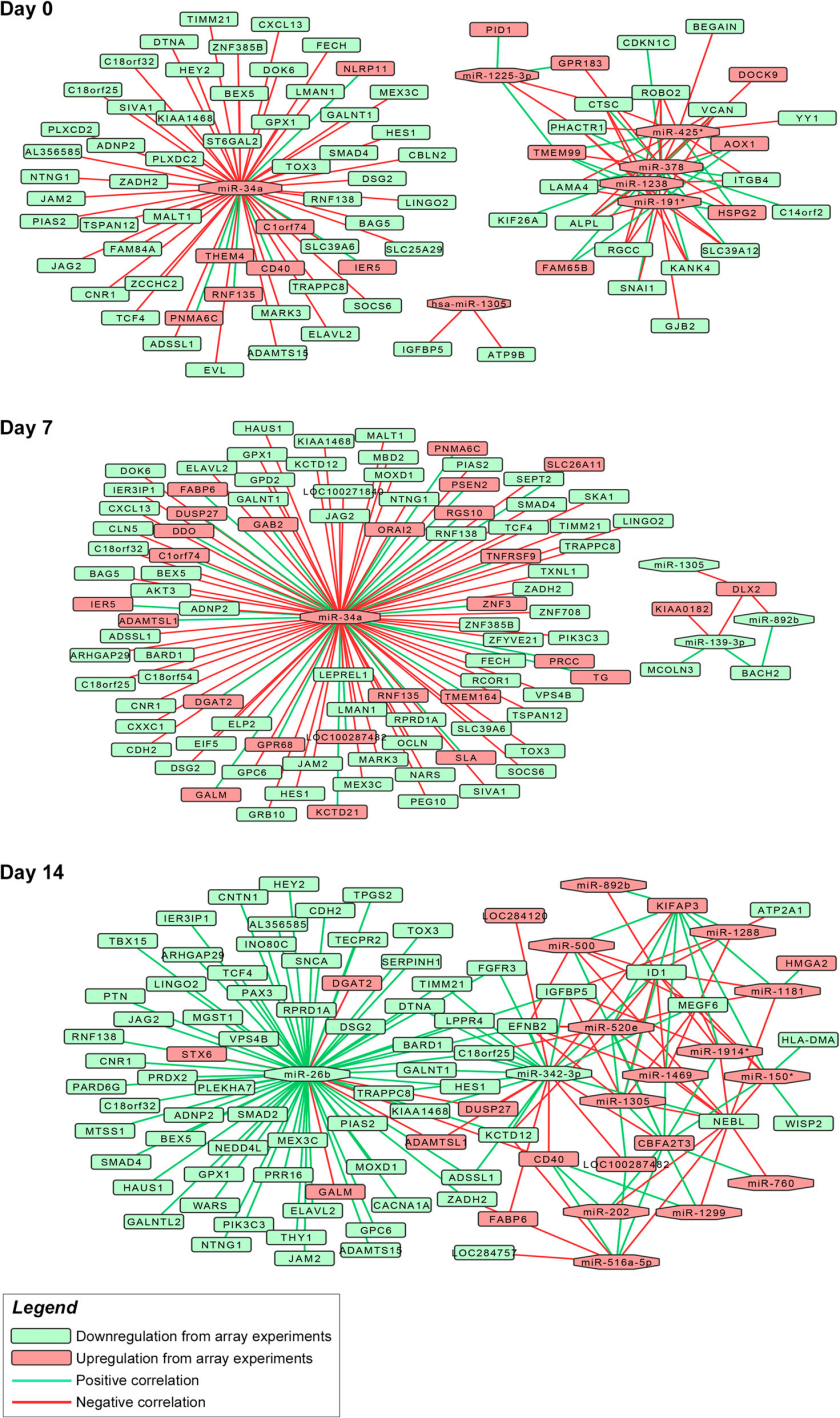
Network representation of significantly correlated miRNA: gene couples. At initial (day 0) and middle (day 7) phases of differentiation miR-34a shows the highest number of correlated probes, instead miR-26b shows the highest number at an advanced stage of differentiation (day 14). Interestingly, miR-34a in particular seems to have a distinct action because of the completely separated network compared to the other miRs

Associations between miRNAs/mRNA were subsequently analyzed by correlative expression analysis. We plotted the frequency distribution of the global correlations across the 3 time points (Additional file 8: Figure S4). Notably, the 3 distributions have 3 distinct shapes and basically deviate from a normal distribution but related frequencies of *p*-values are in line with previously published studies [36]. On a total number of 6954 correlations, 510 (7.3 %) are significant (|r| ≥ 0.7 and *p*-value <0.05) (Additional file 9: Table S5), including both the positive and negative (see Table 1 for details of positive and negative percentages at the 3 time points). Since the analysis was performed at probe level, a total of 52 significant correlations of miRNA:gene were predicted more than once. In detail, we identified 159, 136, and 215 significant correlations at days 0, 7, and 14. The number of significant correlations for each microRNA is strongly heterogeneous (range: 4–207, mean: 23, median: 9). miR-34a show the highest number of negatively correlated probes (n = 70). Positive and significant correlations are 25.2 % and 22.1 % at days 0 and 7 respectively, while this percentage almost triplicates at day 14 (67.4 %) where a single miR, miR-26b, holds the majority of positive correlations (86/145) (Additional file 10: Figure S5). Among negative and significant correlations (70/215), we observed a strong correlation (−0.903) between ID1, one of the main down-stream targets of TGFbeta signaling [37], and miR-520e, a microRNA belonging to the miR-373/520 family: its over-expression has been recently described to have a tumor suppressive role in breast cancer, also by down-regulation of the TGFbeta signaling [38]. To further define interactions among miRNAs and related targets, we represented the resulting network of miRNA:gene by Cytoscape. At basal condition, we observed two main distinct subnetworks (Fig. 5) where part of the genes (56, 68 %) show a perfect star topology with miR-34a (network centralization = 1). The rest of genes are principally interconnected across 5 of 6 remaining miRs (network centralization = 0.552). To verify that the two separate networks were only partially related to the parameters we used for correlation, we tested less stringent combinations of Pearson’s score and *p*-value (data not shown). However, due to the very limited overlap between the two networks (7.5 % at the most) and the consequent increase in the number of false positives, we maintained the initial thresholds to optimize information. The presence of two separated subnetworks suggests a separate mechanism of action of miR-34a respect to the other miRs. miR-34a centrality is more evident at day 7, where almost all correlations concentrate on miR-34a itself. At advanced differentiation stages we observed a more interconnected mesh-like network, although miR-26b polarizes the majority of correlations.

Finally, to define the most interesting couples miR:target, intersection of prediction and correlation approaches were used, identifying 62 couples miR:gene (Table 3). This analysis strongly reduced the number of miRs with a potential to regulate the system, only 9 out of the 22 differentially expressed microRNAs have at least one target significantly predicted and correlated. Five miRs have one single target gene (miR-202: CBFA2T3, miR-500: NEBL, miR-378: KIF26A, miR-892b: BACH2, miR-1305:ID1), with a restricted impact on gene expression modulation. Among the remaining 4 miRs (miR-26b, miR-34a, miR-342-3p, miR-520e), attention still points to miR-34a, which has the highest number (24) of predicted and correlated genes. Most correlations are negative (17/24) and, as expected, involve genes of TGFbeta pathway, such as SMAD4 (r = −0.904), AKT3 (r = −0.909) and GRB10 (r = −0.842). In contrast, the most down-regulated miRNAs, miR-342-3p and miR-26b, showed a prevalence of positive correlations with their targets (respectively 8/9 and 20/22), which indicate a more complex and possible indirect relationship with the regulated genes.

**Table 3:** Couples miRNA:gene after intersection of correlation and prediction approaches. There are 62 couples, where miR-34a shows the highest number of putative negatively correlated genes. When multiple probes per gene are significant, the most correlated is shown

		Correlation					Correlation		
miR name	Gene symbol	Day 0	Day 7	Day 14	miR name	Gene symbol	Day 0	Day 7	Day 14
hsa-miR-1305	ID1	*ns*	*ns*	−0.886	hsa-miR-26b	ADAMTSL1	*ns*	*ns*	−0.929
hsa-miR-202	CBFA2T3	*ns*	*ns*	0.931	hsa-miR-26b	ADNP2	*ns*	*ns*	0.94
hsa-miR-34a	AKT3	*ns*	−0.909	*ns*	hsa-miR-26b	ARHGAP29	*ns*	*ns*	0.924
hsa-miR-34a	ARHGAP29	*ns*	−0.927	*ns*	hsa-miR-26b	C18orf25	*ns*	*ns*	0.918
hsa-miR-34a	C18orf25	−0.945	−0.917	*ns*	hsa-miR-26b	CDH2	*ns*	*ns*	0.968
hsa-miR-34a	C1orf74	0.827	0.827	*ns*	hsa-miR-26b	CNR1	*ns*	*ns*	0.844
hsa-miR-34a	CD40	0.945	*ns*	*ns*	hsa-miR-26b	DTNA	*ns*	*ns*	0.908
hsa-miR-34a	DOK6	−0.885	−0.885	*ns*	hsa-miR-26b	ELAVL2	*ns*	*ns*	0.885
hsa-miR-34a	DTNA	−0.892	*ns*	*ns*	hsa-miR-26b	HES1	*ns*	*ns*	0.909
hsa-miR-34a	GALM	*ns*	0.815	*ns*	hsa-miR-26b	HEY2	*ns*	*ns*	0.94
hsa-miR-34a	GRB10	*ns*	−0.842	*ns*	hsa-miR-26b	JAM2	*ns*	*ns*	0.9
hsa-miR-34a	KCTD12	*ns*	−0.87	*ns*	hsa-miR-26b	KIAA1468	*ns*	*ns*	0.917
hsa-miR-34a	KCTD21	*ns*	0.813	*ns*	hsa-miR-26b	MEX3C	*ns*	*ns*	0.99
hsa-miR-34a	LMAN1	−0.884	−0.88	*ns*	hsa-miR-26b	NEDD4L	*ns*	*ns*	0.883
hsa-miR-34a	MEX3C	−0.938	−0.938	*ns*	hsa-miR-26b	NTNG1	*ns*	*ns*	0.907
hsa-miR-34a	NTNG1	−0.856	−0.856	*ns*	hsa-miR-26b	PLEKHA7	*ns*	*ns*	0.863
hsa-miR-34a	NTNG1	*ns*	−0.856	*ns*	hsa-miR-26b	RNF138	*ns*	*ns*	0.829
hsa-miR-34a	PEG10	*ns*	−0.941	*ns*	hsa-miR-26b	SMAD2	*ns*	*ns*	0.816
hsa-miR-34a	RPRD1A	*ns*	−0.877	*ns*	hsa-miR-26b	SMAD4	*ns*	*ns*	0.933
hsa-miR-34a	SMAD4	−0.904	−0.904	*ns*	hsa-miR-26b	STX6	*ns*	*ns*	−0.858
hsa-miR-34a	SOCS6	−0.982	−0.994	*ns*	hsa-miR-26b	TECPR2	*ns*	*ns*	0.885
hsa-miR-34a	TMEM164	*ns*	0.88	*ns*	hsa-miR-26b	VPS4B	*ns*	*ns*	0.864
hsa-miR-34a	TNFRSF9	*ns*	0.882	*ns*	hsa-miR-342-3p	BARD1	*ns*	*ns*	0.835
hsa-miR-34a	TXNL1	*ns*	−0.931	*ns*	hsa-miR-342-3p	C18orf25	*ns*	*ns*	0.934
hsa-miR-34a	ZFYVE21	*ns*	−0.878	*ns*	hsa-miR-342-3p	EFNB2	*ns*	*ns*	0.866
hsa-miR-34a	ZNF3	*ns*	0.823	*ns*	hsa-miR-342-3p	IGFBP5	*ns*	*ns*	0.897
hsa-miR-378	KIF26A	0.848	*ns*	*ns*	hsa-miR-342-3p	KCTD12	*ns*	*ns*	0.923
hsa-miR-500	NEBL	*ns*	*ns*	−0.903	hsa-miR-342-3p	KIAA1468	*ns*	*ns*	0.834
hsa-miR-520e	C18orf25	*ns*	*ns*	−0.823	hsa-miR-342-3p	KIFAP3	*ns*	*ns*	−0.903
hsa-miR-520e	EFNB2	*ns*	*ns*	−0.813	hsa-miR-342-3p	LPPR4	*ns*	*ns*	0.872
hsa-miR-892b	BACH2	*ns*	0.813	*ns*	hsa-miR-342-3p	MEGF6	*ns*	*ns*	0.951

*ns* = not significant

## Discussion

Etiology of OS still remains unclear although recent papers have related this tumor to a molecular blockade of the normal process of osteoblast differentiation [18]. OS cells are thought to derive from mesenchymal stem cells already committed towards osteblast differentiation and thus displaying features of osteo-progenitors [26]. Transformation hampers progression of malignant cells towards terminal osteoblastic differentiation while maintaining cell proliferation.

High-throughput screening techniques appear the most refined tool to identify pivotal players in complex biological processes: combination of different approaches to integrate several platforms can help better explore their fine tuning activity. Some authors have recently integrated a considerable number of high-throughput data from OS cell lines, either by miRNA vs mRNA expression profiles [39, 40] or vs CGH arrays [41] or by protein arrays [42], identifying miR-17/92 cluster [39] or TGFbeta pathway [42] as crucial mediators. However, all these studies focalized on the events at basal conditions and their expression during the differentiative process was not explored.

Besides alterations in Rb, p53 [18], and oncogene MET pathways, CD99 transmembrane antigen was also found to regulate differentiation of OS cells [26, 28]. In this paper we used CD99 transfected OS cells to explore the temporal transcriptional changes that couple with malignancy reversal and modulation of differentiation. CD99 is generally lost in OS cells and, when its expression is restored, the molecule switches cells from proliferation status to cell cycle withdrawal, favoring the achievement of a terminally osteoblast-differentiated phenotype, thus resembling the fate of mature osteoblasts [28]. This phenotype is confirmed by PCA meta-analysis, which shows the shift of Saos-2 cell genetic profile towards normal osteoblasts when CD99 is re-expressed. Gene expression analysis indicated that CD99 forced expression induced broad gene expression down-regulation: almost 80 % of genes showed decreased expression in CD99 transfected cells. Enrichment analysis revealed a significant modulation in Epithelial to Mesenchymal Transition (EMT), apoptosis and TGFbeta signaling pathways.

EMT has received considerable attention in the last years as a paradigm to explain invasive and metastatic behavior of cancer cells. Firstly described in carcinomas, the hallmarks of this program are the disruption of cell adhesion structures between adjacent cells, a dramatic remodeling of the cytoskeleton, and the acquisition of a mesenchymal phenotype. Reduced expression of epithelial markers, such as E-cadherin, and a simultaneous increase in mesenchymal marker expression, such as N-cadherin and vimentin characterize EMT, whose master regulators, *SNAIL1* and *SNAIL2* are direct transcriptional targets of the TGFbeta pathway SMADs mediators [43]. OS is characterized by the expression of EMT-related transcription factors, which are involved in the complex pathogenesis of the tumor [44]. Over-expression of CD99 determines a down-regulation of mesenchymal markers, such as fibronectin, and the transcription factor snail1. In keeping with the less malignant behavior, Sa/CD99 OS cells also exhibited down-modulation in genes belonging to TGFbeta signaling, a pathway that plays fundamental roles in carcinogenesis. TGFbeta, one of the most abundant growth factors stored and released by bone, is known to regulate a broad range of biological processes, including cell proliferation, cell survival, cell differentiation, cell migration, production of extracellular matrix molecules and regulation of cell stemness [45]. The combined actions of these cellular responses mediate the global effects of TGF-beta pathway on cancer, immune responses, angiogenesis, wound healing, development, and bone formation. In cancer, several studies have clearly demonstrated that TGFbeta signaling pathway can either foster or suppress tumor progression [46, 47]: depending on the cellular context and the type of TGF-beta signaling pathway that is initiated (Smad-dependent or Smad-independent pathway), the cell is directed to undergo either proliferation, differentiation or apoptosis. In the bone environment TGFbeta signaling is reported to inhibit osteoblast differentiation [48] while inducing proliferation and migration in OS cells [49, 50]. Together with hypoxia, TGFbeta has also been shown as an important element that prompts OS cells toward cancer stem cell phenotype [51]. In addition, Yang and Franchi et al. also found higher TGFbeta1 expression in the patients with high-grade osteosarcoma and lung metastasis [52, 53], indicating that TGF-beta signaling promoted the chemoresistance, tumorigenicity, and metastatic potential of OS. Decreased expression of genes belonging to TGFbeta signaling in the Sa/CD99 OS, which are reverted in malignancy and prone to differentiation, is thus consistent with the potential value of therapies targeting TGFbeta signaling. Reversing the tumorigenicity of OS cells and placing them back on the road to normal osteoblasts differentiation means not only the induction of progressive loss of proliferative capacities but generation of apoptosis, which is part of the program cell fate toward terminal osteoblast differentiation to osteocytes. According to these processes, network analysis of genes modulated in Sa/CD99 cells identified two core hubs composed of AKT1 and SMAD 2/4 proteins. In particular, SMAD4, one of the leading mediators of TGFbeta signaling [54], resulted to be the main hub with the highest number of connected genes (8/16). This protein has been found to have an oncogenic role in sarcomas [55] and in glioblastoma [56], while SMAD4 silencing induced growth inhibition and apoptosis in rhabdomyosarcoma cells [57]. Observation of the network analysis depicted a changeable entourage of important mediators that act at different stages. In particular, silencing of ID1, which interacts directly with SMAD4 at day 14, is essential for activation of terminal differentiation [37], while SMAD2, a partner of SMAD4 in TGFbeta signaling, enhances SMAD4 expression and suppresses osteocalcin [58], a marker of late osteoblast differentiation. SMAD2 is also expressed in the majority (85 %) of OS clinical samples [59], and its repression by microRNA mimics inhibits proliferation and invasion in OS cells [50]. The other component of the hub, AKT1 plays an important role in proliferation, survival, migration and metastatization of many cancers including OS [60]. Its widespread activation has been observed in OS patients with pulmonary metastatic disease [60], and AKT1 inhibition by Akt-siRNA or allosteric specific inhibitors was found to decrease cell migration and/or inhibit proliferation in several OS experimental models [61, 62], supporting the therapeutic attractiveness of AKT-targeted inhibitors. Besides direct activation, multiple studies have also suggested the existence of indirect mechanisms for TGFbeta activation of PI3K/AKT, where TGFbeta may act in concert with other stimuli. On the other hand, PI3K/AKT antagonizes TGFbeta-induced cytostatic responses and causes the shift in TGFbeta/SMAD signaling to its tumor-promoting mode during malignant tumor progression, thus indicating the existence of a signaling interplay between TGFbeta and PI3K/AKT pathways.

Taken together, annotation analysis of Sa/CD99 gene expression with respect to parental cells clearly identifies in down-regulation of TGFbeta/Smad4/Akt signaling a crucial event in the reversion of OS malignancy. Interestingly, miRNAs expression profiling also indicated a modulation of miRNAs that converge on TGFbeta pathway. A general miRNA up-regulation was observed after CD99 transfection. A signature of 22 modulated miRNAs was defined and their expression correlated with significantly modulated genes to detect important associations at each phase of cell differentiation. Network analysis identified in miR-34a and miR-26b the two main regulators in early (day 0 and day 7) or in late (day 14) phases of differentiation. These two miRs exhibit an opposite mechanism in regulating their respective targets: miR-26 mainly shows a direct correlation, which introduces to an indirect and difficult to unravel mechanism of regulation, whereas miR-34a displays a canonical down-regulation of its targets.

Enrichment analysis on predicted targets of significantly modulated miRNAs confirmed that TGFbeta signaling and apoptosis-related mechanisms could be miRNA-driven. In particular, miR-34a emerges as the main putative modulator of several genes of TGFbeta signaling. In our model, SMAD4 resulted to be the best candidate target at transcriptional level, in line with regulation of SMAD4 by miR-34a that has been recently shown in glioblastoma [56]. miR-34a is a well-known oncosuppressor miRNA found to induce cell-cycle arrest and apoptosis thorough negative regulation of proteins directly involved in the regulation of cell proliferation and/or cell death, such as E2F, cyclin D1, CDK4, CDK6, cyclin E2, and bcl-2 [63, 64]. miR-34, whose expression is generally reduced in most tumors [33] including OS [65], also displays a role in osteogenic differentiation [66]. Our results further support these evidences, indicating miR-34a as a leader player in the reversal of malignancy and reactivation of differentiation of OS cells by TGFbeta signaling down-modulation.

Complexity of the genetic landscape in OS cells together with its rarity make any targeted therapy difficult to be defined. In this context, the multiple approaches here adopted may represent a powerful tool to unravel and better characterize the genetic background associated with malignant phenotype of this tumor, thus offering identification of critical hubs for the design of differentiative therapeutic strategies.

## Conclusions

Our intent was to define the transcriptional modifications that characterize reversion of malignancy and induction of terminal osteoblast differentiation in OS. A global gene down-regulation was observed across the 14 days of in vitro differentiation and down-modulation of the TGFbeta signaling pathway, together with involvement of several important mediators like AKT1 and SMADs proteins, were defined as crucial events. The use of multiple analyses supported the interactions between miRNome and transcriptome and helped to define miRNAs impact on gene down-modulation. miR-34a was clearly identified as key regulator at initial and middle phases of OS differentiation toward osteoblast. To our knowledge, this is the first study where deciphering of the miRNAs role in the differentiative block of OS is tracked in time. miR-34a up-regulation followed by TGFbeta/SMAD4 signaling inhibition appeared as two crucial players able to induce malignant reversion and osteogenic differentiation of OS cells. These new insights could drive future efforts to investigate the relevance of miR-34a and TGFbeta signaling as potential targets for innovative therapeutic strategies against OS.

## Methods

### Cell lines

OS cell line Saos-2 was obtained from American Type Culture Collection (Manassas, VA, Rockville, MD). Stable transfectants expressing CD99 were obtained from Saos-2 cell line (Sa/CD99wt22, Sa/CD99wt36) by using calcium-phosphate transfection method [28] and tested for mycoplasma contamination every three months (last control September 2014) by PCR Mycoplasma detection Set (Takara Bio Inc., Shiga, Japan).

Cells were routinely cultured in Iscove’s modified Dulbecco’s medium (IMDM), supplemented with 100 U/ml penicillin, 100 μg/ml streptomycin, and 10 % inactivated fetal bovine serum (FBS) and maintained at 37 °C in a humidified 5 % CO_2_ atmosphere. Transfectants were selected in IMDM containing 10 % FBS and 500 μg/ml neomycin.

### Osteoblast differentiation

Four days after seeding cells were exposed to specific osteogenic medium, IMDM supplemented with 2 % FBS, 5 mM β-glycerophosphate and 50 μg/mL ascorbic acid (Sigma-Aldrich, St. Louis, MO) and maintained in differentiative conditions up to 14 days. Medium was renewed every 4 days. Cultures were harvested at various time points to collect RNA. Total RNA was extracted by the TRIzol extraction kit (Life Technologies, Grand Island, NY). Quality and quantity of RNA samples were assessed with NanoDrop analysis (NanoDrop Technologies). Expression of CD99 was verified by real-time PCR at basal and differentiative conditions in all profiled samples (Additional file 11: Figure S6).

### Microarray hybridization

Gene expression of Saos-2 and Sa/CD99 cells was profiled by Affymetrix (Santa Clara, CA, USA) GeneChip Human Genome U133A plus 2 according to manufacturer’s instructions and scanned by Affymetrix Scanner 3000 7G to obtain raw data. miRNAs profile was analyzed by Agilent (Santa Clara, CA, USA) Human miRNA microarray platform (v.3) according to manufacturer’s instructions, scanned with Agilent Scanner G2505 and analyzed by Feature Extraction (v10.5) software to obtain raw data. All raw data were inspected for visual and technical artifacts by Bioconductor (v 2.9) packages (*simpleaffy* [67], *affyPLM* and *AgiMicroRNA* [68] for miRNA data) on R (v. 2.15) and were considered of good quality. (see Additional file 12: Figure S7 for density plots of both gene and miRNA expression data, and Additional file 13: Table S6 for gene expression quality metrics). Two samples for Saos-2 and two for Sa/CD99 cells were profiled for each time point, for a total of 12 samples for either gene or miRNA expression profiling.

### mRNA and miRNA expression profiling

All microarray analyses were performed by Bioconductor packages on R. Gene expression data were quantified and normalized by *rma* [69] algorithm and log2 transformed. Probes with low expression (signal intensity of the probe ≤ 100 of absolute values in at least 75 % of samples) and low IQR, Inter-Quartile Range (IQR of log2 signals ≤ median of IQR across all samples) were filtered out. Differentially expressed genes between Sa/CD99 vs Saos-2 were detected using *limma* [70] package and corrected by FDR according to Benjamini and Hochberg multiple test correction. Genes were considered significant when logFC ≥ 0.585 (logFC, log fold change of absolute normalized values), corresponding to a fold change of 1.5, and *p*-value ≤ 0.05 after multiple test correction. Only probes matching well annotated genes according to Affymetrix HG_U133 Plus 2.0 array annotation and recovered by *hgu133plus2.db* Bioconductor package were preserved. MicroRNA expression data were quantified, log2 transformed and normalized in R using *AgiMicroRna* package [68], that uses an adaptation of rma method for microRNA data. Low or not expressed miRs were filtered out and remaining probes were tested for differential expression using *limma* modified *t*-test: miR was considered significant when logFC ≥ 0.485, corresponding to a fold change of 1.4, and *p*-value ≤ 0.01 in transfected vs parental cell lines. Microarray data are available at GEO database with SuperSeries accession number GSE61930.

### Correlations between miRNAs and genes profiling

Correlation was calculated between expression of miRNAs vs gene probes differentially expressed in at least 1 on 3 time points. Analysis was performed on normalized log2 expression data and considered significant when: |r| ≥ 0.7 and p ≤ 0.05, where r is the score according to Pearson’s correlation and p is the asymptotic *p*-value. Calculation was performed by R scripts using library *Hmisc*. For gene expression data, correlation was preserved at probe level, thus multiple significant correlations per gene may exist. Both positive and negative correlations were considered with putative biological relevance [10]. No multiple test correction was performed; to control type I error without reducing statistical power we opted for the use of an high Pearson’s r score.

### miRNA target prediction

Genes were considered putative target of microRNA when reported in at least 1 of the following databases: Targetscan (v. 6.0), miRanda (v. 3.0), Diana MicroT (v. 3.0), PicTar (v. 4-way). All predictions were downloaded from their respective web sites except for PicTar from UCSC genome browser (v. NCBI35/hg17). Only differentially expressed genes and miRs were taken into account for microRNA target prediction analysis. No score threshold was used for target prediction.

### Enrichment and network analyses

Enrichment analysis of pathways was performed using MetaCore in GeneGO (Thomson Reuters, New York, NY, USA) program. Biological pathways were defined according to “GeneGO Pathway Maps” manually curated database. Pathways were tested for significance by modified Fisher’s Exact Test and were corrected by FDR multiple test correction according to the Benjamini and Hochberg method, considered significant when the corrected *p*-value of enrichment was ≤ 0.1. Network analysis of biologically-related terms was performed with the direct interaction method in GeneGO, were an edge connecting two genes indicates their direct biological relation according to MetaCore database, which includes manually-curated database of human gene and protein interactions, built according to published literature. Network analysis was performed on genes differentially expressed at all time points. Background for statistical analysis by GeneGO is composed of the array gene list. Graphical representation of miRNA-gene from correlation and prediction analyses was performed using Cytoscape (v.3.0) [71].

### Meta-analysis

Expression data of human osteoblast derived from bone marrow hMSCs were recovered in GEO database with ID GSE9451 [31]. Expression data from osteosarcoma and mesenchymal cell lines were *rma* normalized together and log2 transformed before analysis. No further batch correction was performed. Meta-analysis was performed using *made4* package [72] on R. Expression data of differentially expressed genes in Sa/CD99 vs Saos-2 at day 0 or day 14 was used for PCA analysis for day 0 or day 14 respectively. The “pca” method for “ord” (ordination method) function and default settings as from *made4* introduction file were used.

## Additional files

Additional file 1: Table S1. Genes differentially expressed in Sa/CD99 vs. Saos-2 cells in at least one time point. Additional file 2: Figure S1. Volcano plot of gene (a) and miRNA (b) expression data: for both experiments, we marked out genes and miRNA differentially expressed in Sa/CD99 vs Saos-2 at day 0 (red), day 7 (green) and day 14 (blu). Additional file 3: Table S2. Thirty most significant pathways after enrichment analysis on differentially expressed genes. Additional file 4: Figure S2. Network analysis of differentially expressed genes at each single time point. Genes from the core network (Fig. 3) are circled in red. Additional file 5: Table S3. Euclidean distances of the eigenvector from PCA analysis for Sa/CD99 transfectants, Saos-2 parental and Osteoblast from BM-MSC cells. Distance is calculated on the mean of eigenvectors for each cell type. Additional file 6: Table S4. Couples of significant gene-miR from prediction approach. Additional file 7: Figure S3. Enrichment analysis of biologically modulated pathways on modulated predicted targets. Additional file 8: Figure S4. Frequency histogram of Pearson’s correlation scores (on left) and of related *p*-values (on right) at the three time points for differentially expressed genes and miRNAs. Dashed lines indicate the threshold (|r| ≥ 0.7 and *p*-value ≤ 0.05) used to consider significant a correlation. Additional file 9: Table S5. Couples of significant gene-miR from correlation approach. Additional file 10: Figure S5. Percentage of negative and positive correlations at each time point and at all time points. Additional file 11: Figure S6. Expression levels of CD99 were verified by real-time PCR in parental Saos-2 and both clones for all three days analyzed by expression profiling. Additional file 12: Figure S7. Density plots of gene and miRNA expression data for all arrays, used for quality control. Additional file 13: Table S6. Quality control parameters of gene expression arrays are shown. All values respect suggestion of Affymetrix quality control (for details see *simple affy* package vignettes).

## Abbreviations

miRNAs or miRs: microRNAs
OS: Osteosarcoma
hMSCs: Human mesenchymal stem cells
EMT: Epithelial-mesenchymal transition
