# Supplementary material for: Integrated approaches to miRNAs target definition: time-series analysis in an osteosarcoma differentiative model
Source: BMC Med Genomics. 2015 Jun 30;8:34. doi: 10.1186/s12920-015-0106-0 (PMC4486310; doi:10.1186/s12920-015-0106-0)
Supplement: Additional file 2: Figure S1. — Volcano plot of gene (a) and miRNA (b) expression data: for both experiments, we marked out genes and miRNA differentially expressed in Sa/CD99 vs Saos-2 at day 0 (red), day 7 (green) and day 14 (blu). [file 12920_2015_106_MOESM2_ESM.pdf]

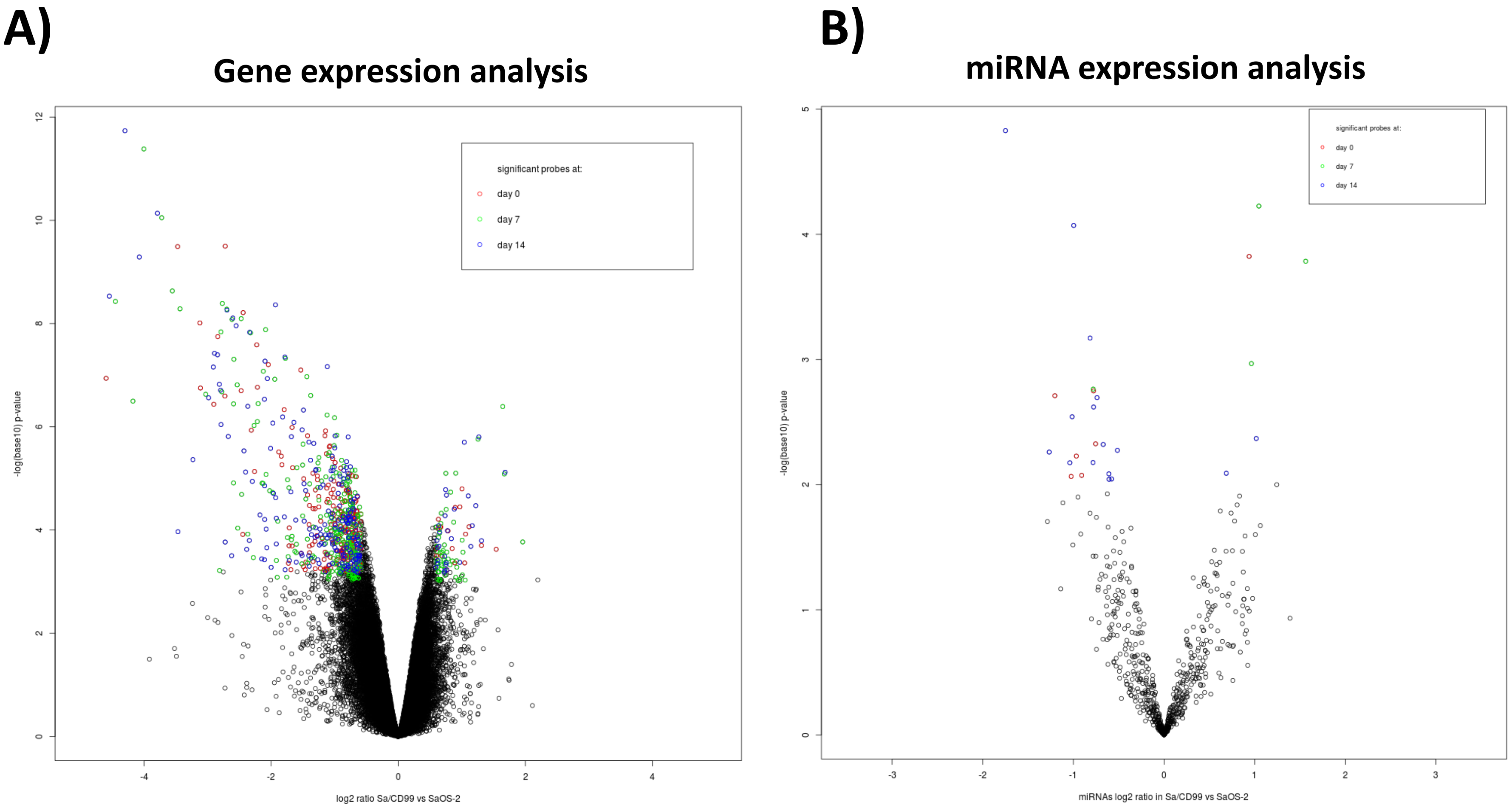

Supplementary Figure S1.

Volcano plot of gene (a) and miRNA (b) expression data: for both experiments, we marked out genes and miRNA differentially expressed in Sa/CD99 vs Saos-2 at day 0 (red), day 7 (green) and day 14 (blu).
