# Supplementary figures and images for: Integrated approaches to miRNAs target definition: time-series analysis in an osteosarcoma differentiative model
Source: BMC Med Genomics. 2015 Jun 30;8:34. doi: 10.1186/s12920-015-0106-0 (PMC4486310; doi:10.1186/s12920-015-0106-0)

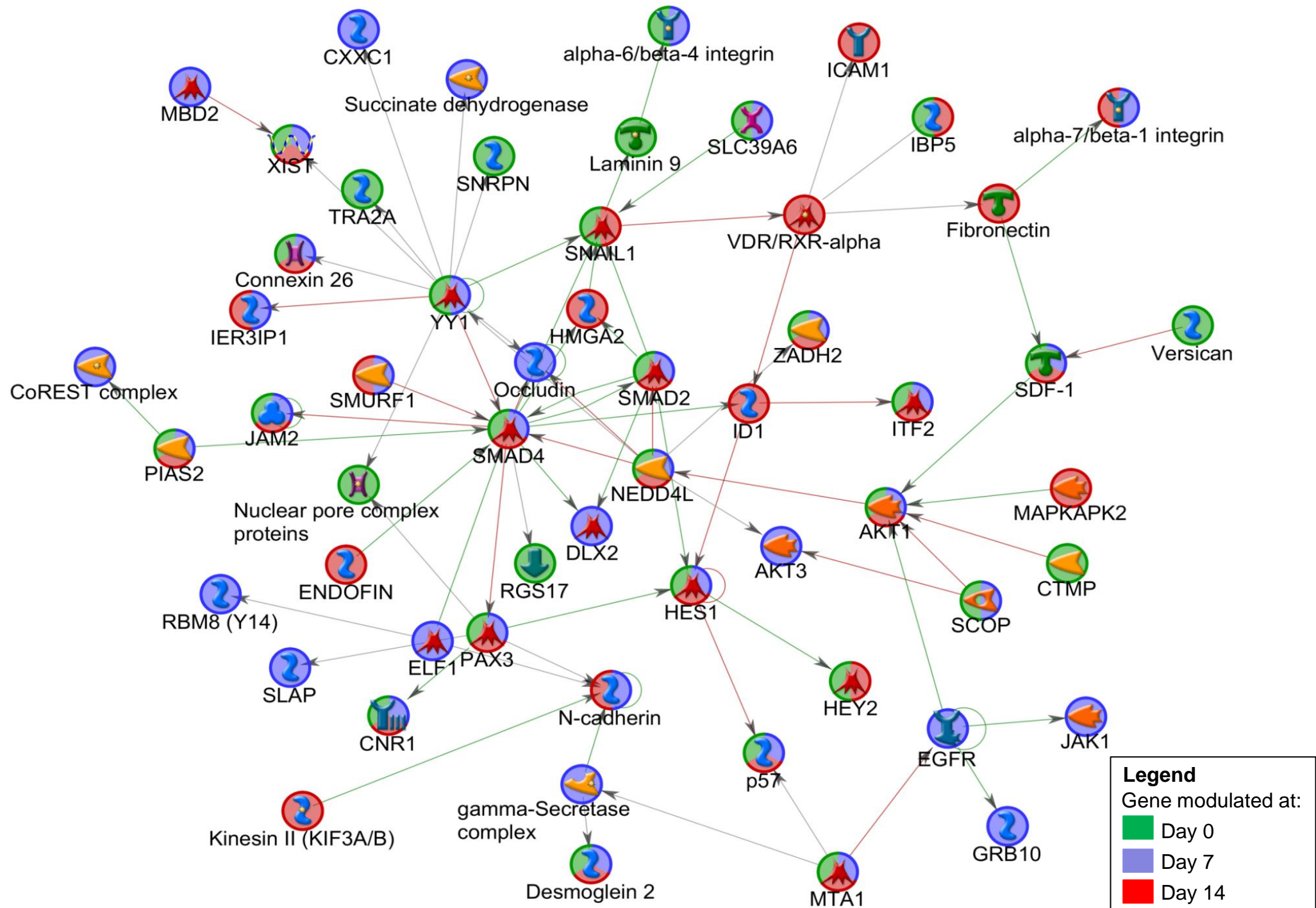

Supplement: Additional file 4: Figure S2. — Network analysis of differentially expressed genes at each single time point. Genes from the core network (Fig. 3) are circled in red. [file 12920_2015_106_MOESM4_ESM.pdf]
