# Supplementary material for: Integrated approaches to miRNAs target definition: time-series analysis in an osteosarcoma differentiative model
Source: BMC Med Genomics. 2015 Jun 30;8:34. doi: 10.1186/s12920-015-0106-0 (PMC4486310; doi:10.1186/s12920-015-0106-0)
Supplement: Additional file 7: Figure S3. — Enrichment analysis of biologically modulated pathways on modulated predicted targets. [file 12920_2015_106_MOESM7_ESM.pdf]

#	Maps	0 1 2 3 4 5 -log(pValue)	pValue	min(pValue)	Ratio
1	Development_TGF-beta-dependent induction of EMT via SMADs		1.838e-7<br>1.982e-4<br>7.635e-3	1.838e-7	6/35
2	Development_HGF signaling pathway		5.059e-3<br>1.555e-5<br>1.349e-2	1.555e-5	4/47
3	Development_TGF-beta receptor signaling		6.020e-3<br>1.994e-5<br>3.442e-5	1.994e-5	5/50
4	Development_WNT signaling pathway. Part 2		6.230e-4<br>2.518e-5<br>1.014e-3	2.518e-5	4/53
5	Cytoskeleton remodeling_TGF, WNT and cytoskeletal remodeling		1.365e-3<br>2.749e-5<br>7.614e-4	2.749e-5	6/111
6	Apoptosis and survival_Apoptotic Activin A signaling		8.062e-4<br>7.121e-5<br>1.070e-4	7.121e-5	3/25
7	Immune response_IL-10 signaling pathway		1.769e-1<br>8.273e-2<br>1.206e-4	1.206e-4	3/26
8	Normal and pathological TGF-beta-mediated regulation of cell proliferation		2.486e-2<br>1.659e-4<br>6.807e-3	1.659e-4	3/33
9	Immune response_IL-4 - antiapoptotic action		2.012e-1<br>4.342e-3<br>1.864e-4	1.864e-4	4/30
10	Signal transduction_Activin A signaling regulation		1.833e-3<br>5.239e-3<br>2.486e-4	2.486e-4	3/33

Day 0 Day 7 Day 14

Figure S3. Enrichment analysis of biologically modulated pathways on modulated predicted targets .
