# Supplementary material for: Integrated approaches to miRNAs target definition: time-series analysis in an osteosarcoma differentiative model
Source: BMC Med Genomics. 2015 Jun 30;8:34. doi: 10.1186/s12920-015-0106-0 (PMC4486310; doi:10.1186/s12920-015-0106-0)
Supplement: Additional file 8: Figure S4. — Frequency histogram of Pearson’s correlation scores (on left) and of related p-values (on right) at the three time points for differentially expressed genes and miRNAs. Dashed lines indicate the threshold (|r| ≥ 0.7 and p-value ≤ 0.05) used to consider significant a correlation. [file 12920_2015_106_MOESM8_ESM.pdf]

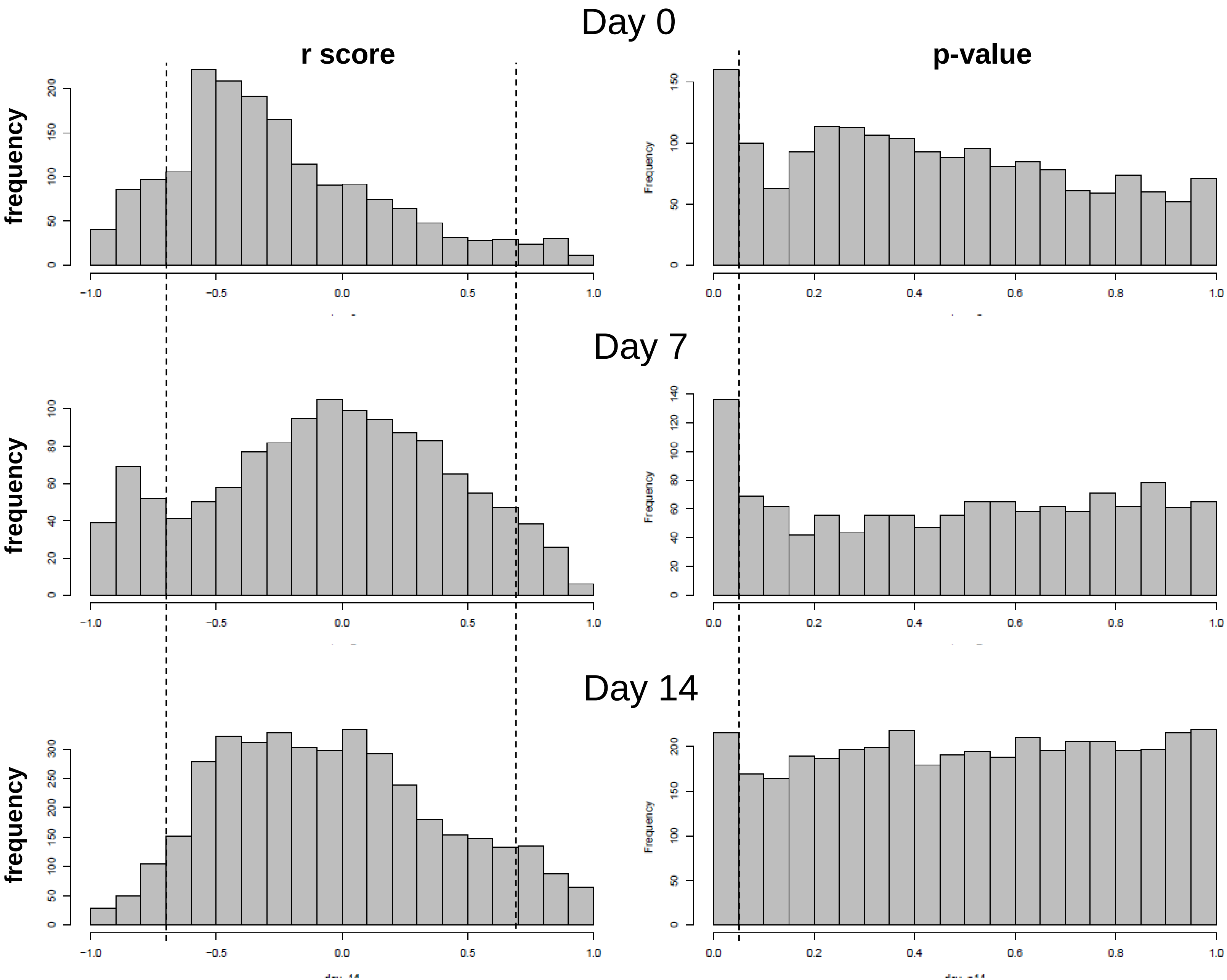

Figure S4. Frequency histogram of Pearson's correlation scores (on left) and of related p-values (on right) at the three time points for differentially expressed genes and miRNAs. Dashed lines indicate the threshold (|r| ≥0.7 and p-value ≤0.05) used to consider significant a correlation.
