# Supplementary material for: Integrated approaches to miRNAs target definition: time-series analysis in an osteosarcoma differentiative model
Source: BMC Med Genomics. 2015 Jun 30;8:34. doi: 10.1186/s12920-015-0106-0 (PMC4486310; doi:10.1186/s12920-015-0106-0)
Supplement: Additional file 11: Figure S6. — Expression levels of CD99 were verified by real-time PCR in parental Saos-2 and both clones for all three days analyzed by expression profiling. [file 12920_2015_106_MOESM11_ESM.pdf]

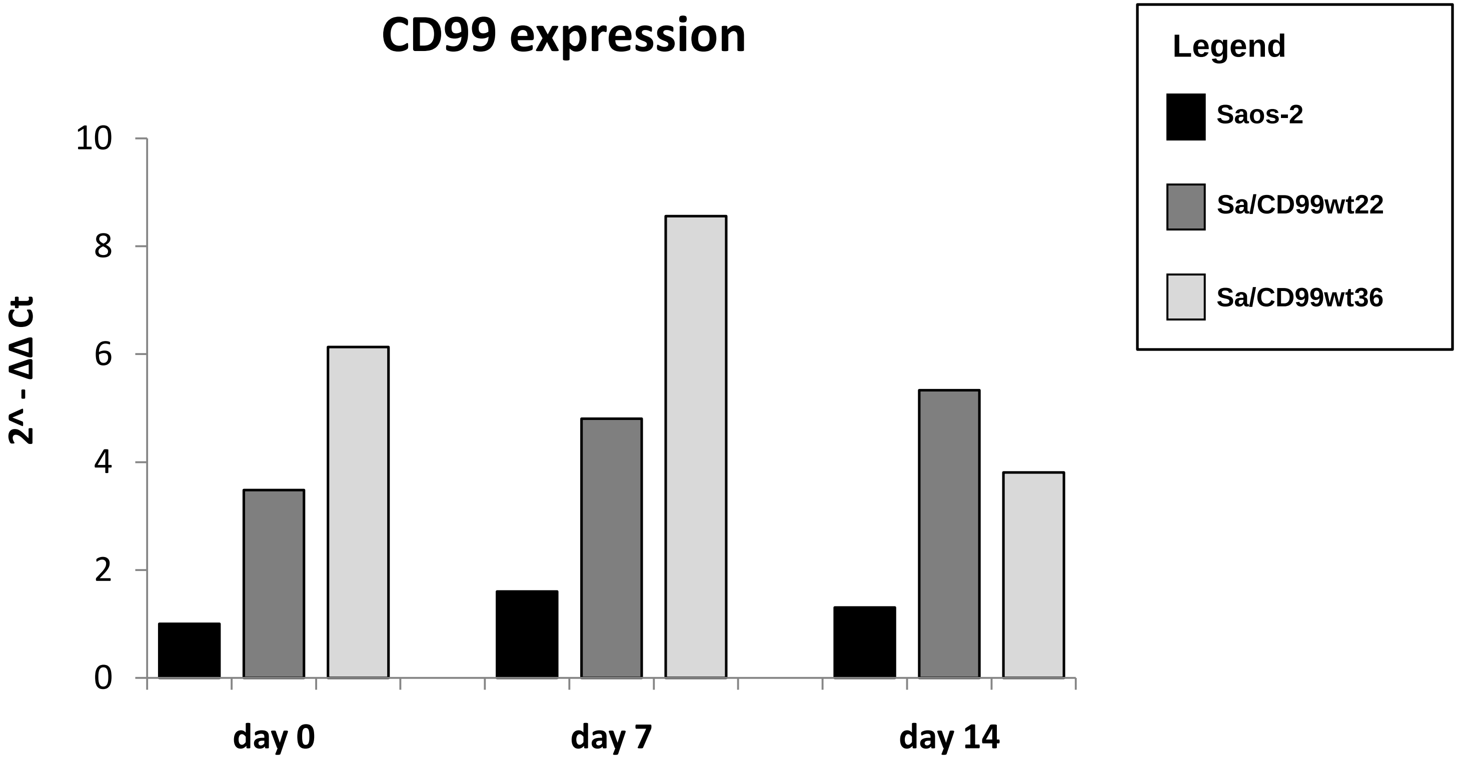

Supplementary Table S6

Expression levels of CD99 were verified by real-time PCR in parental Saos-2 and both clones for all three days analyzed by expression profiling.
