# Supplementary material for: Integrated approaches to miRNAs target definition: time-series analysis in an osteosarcoma differentiative model
Source: BMC Med Genomics. 2015 Jun 30;8:34. doi: 10.1186/s12920-015-0106-0 (PMC4486310; doi:10.1186/s12920-015-0106-0)
Supplement: Additional file 12: Figure S7. — Density plots of gene and miRNA expression data for all arrays, used for quality control. [file 12920_2015_106_MOESM12_ESM.pdf]

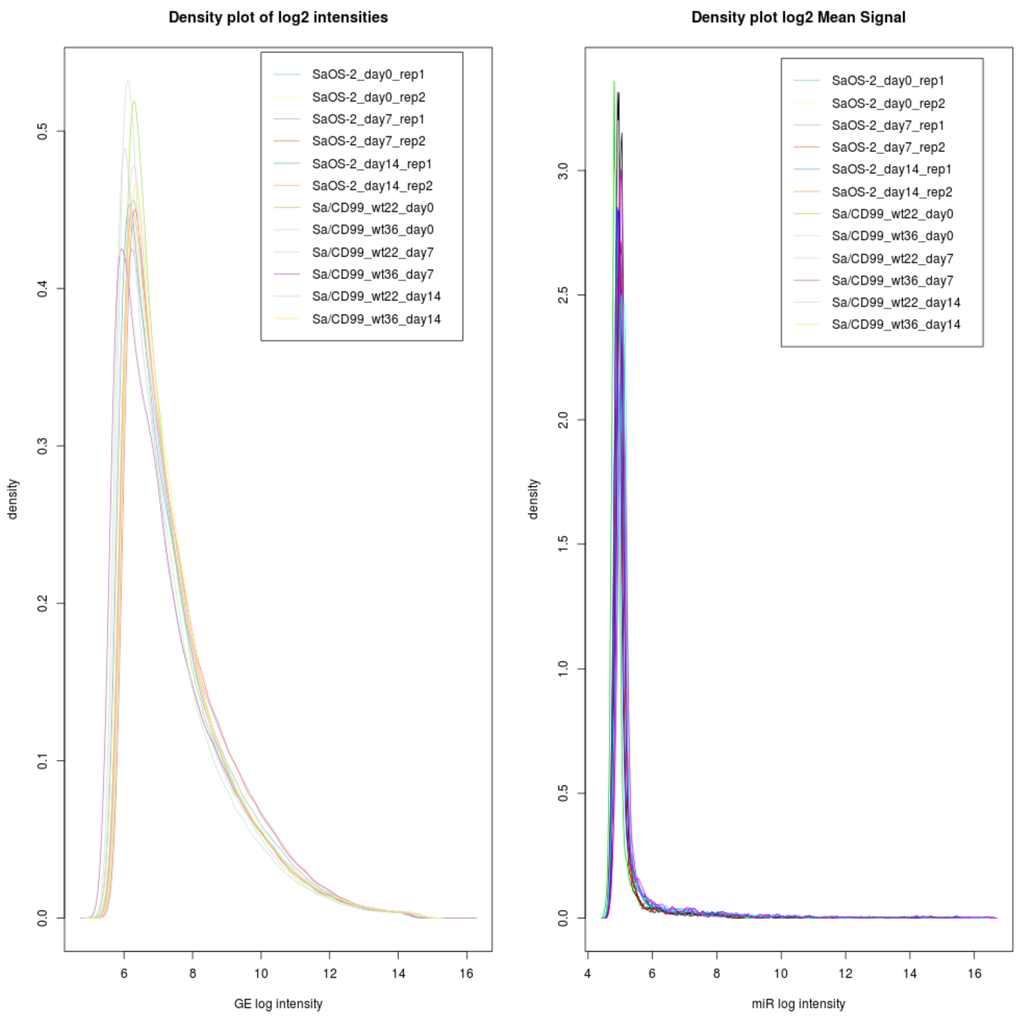

Figure S7:

Density plots of gene and miRNA expression data for all arrays, used for quality control.
